# Isobaric Tags for Relative and Absolute Quantitation Identification of Blood Proteins Relevant to Paroxetine Response in Patients With Major Depressive Disorder

**DOI:** 10.3389/fpsyt.2022.577857

**Published:** 2022-04-18

**Authors:** Chin-Chuen Lin, Hung Su, Jentaie Shiea, Tiao-Lai Huang

**Affiliations:** ^1^Department of Psychiatry, Kaohsiung Chang Gung Memorial Hospital, Chang Gung University College of Medicine, Kaohsiung, Taiwan; ^2^Department of Chemistry, National Sun Yat-sen University, Kaohsiung, Taiwan; ^3^Genomic and Proteomic Core Laboratory, Department of Medical Research, Kaohsiung Chang Gung Memorial Hospital, Kaohsiung, Taiwan

**Keywords:** eukaryotic translation initiation factor 4H (eIF4H), iTRAQ, major depressive disorder, putative hydroxypyruvate isomerase (HYI), RNA binding motif 8A (RBM8A)

## Abstract

**Objectives:**

Isobaric tags for relative and absolute quantitation (iTRAQ) is a proteomic investigation that could be utilized for rapid identification and quantification of proteins, which we would use to identify differentially expressed proteins in treatment responsive patients with major depressive disorder (MDD).

**Methods:**

Six treatment responsive patients of MDD were recruited, and their peripheral blood mononuclear cell (PBMC) were collected before and after 4 weeks of paroxetine treatment. iTRAQ and Mascot search engine were used to detect differentially expressed proteins, which were then validated by Western blot.

**Results:**

Two thousand one hundred and fifty three proteins were screened, and seven proteins showed differences of more than two-fold and 62 proteins with a differences of less than two-fold. Six proteins with commercially available antibodies were identified, and were validated by Western blot in 10 paroxetine responsive MDD patients. Putative hydroxypyruvate isomerase (HYI), eukaryotic translation initiation factor 4H (eIF4H), and RNA binding motif 8A (RBM8A) had statistically significant differences before and after treatment in the validation. Data are available via ProteomeXchange with identifier PXD028947.

**Conclusions:**

By using iTRAQ and Western blot, we were able to identify HYI, eIF4H, and RAM8a to be the potential predictors of paroxetine treatment response in patients with MDD. This finding could help establish future individualized medicine.

## Introduction

Major depression disorder (MDD) is a mental illness characterized by pathological low mood, loss of interest or pleasure, and pessimistic thoughts that negatively impact the quality of life of a significant proportion of the world's population, and has a lifetime incidence ranging from 4.4 to 20% (1). In several decades, many studies have tried to identify the underlying pathogenic mechanisms for MDD (2). However, the pathogenesis of the MDD and related biological regulatory mechanisms are still largely unknown. Therefore, utilization of new technologies to search for potential biomarkers in MDD is mandated.

After the successful deconstruction of the human genome, it was first thought that decoding the gene would be sufficient to unravel the mystery of human brain. However, it was found that genes alone do not necessarily represent the phenotypes, thus entered the post-genome exploration, where the human proteomics program aims at the systematic study of protein expressions.

Earlier proteomic studies frequently utilized cerebrospinal fluid (CSF) or postmortem brain tissues to identify the disease relevant molecules in MDD. The known pathogenesis of MDD involves many brain regions, but most studies focus on one regulatory pathway at a time (3, 4). In clinical population, CSF is challenging to collect, let alone brain tissues. Therefore, investigations of peripheral blood of patients of MDD allowed easier access and flexibility, such as comparing the proteomic changes before and after antidepressant treatment (5). Peripheral blood mononuclear cell (PBMC) has been suggested to be an ideal peripheral meidrum in searching of biomarker (5). Functional lymphatic vessels found in dural sinuses (6) suggested a possible crosstalk between central nervous system (CNS) and the peripheral (7). PBMC has been utilized in both animal (5) and clinical studies of MDD (8, 9).

In the past, we analyzed the peripheral blood of patients with MDD, using acid hydrolysis and modern mass spectrometry, and found that the ratio of transferrin to fibrinogen was significantly different between patients and healthy controls (10, 11). The same technique has been successfully applied to serum samples of patients with schizophrenia, before and after antipsychotic treatment. In that study we found that the serum IgG content changed significantly after risperidone, a frequently prescribed antipsychotic, treatment (12). Furthermore, we also observed that disulfide-isomerase A3 and F-actin-capping protein subunit beta in platelet samples of patients with MDD have higher performance than those of healthy controls using two-dimensional differential gel electrophoresis (2D-DIGE) (13).

2D-DIGE, however, is limited by the range of isoelectric point and molecular weight, and the development of isobaric tags for relative and absolute quantitation (iTRAQ) could be utilized for rapid identification and quantification of proteins. Wang et al. used the iTRAQ method to study relevant proteins in human serum and quickly screened 472 proteins, of which 154 were found to be differentially expressed between patients with depression and healthy conrtols (14). In 2015, Han et al. used iTRAQ technology to study hippocampal postsynaptic density-associated proteins in animal models, screened 1,500 proteins, and found that 74 membrane proteins were differentially expressed (15). Palmfeldt et al. investigated the prefrontal cortices of rat models of depression with iTRAQ and found several pathways that may contribute to the development of new therapeutic directions (16).

Another clinical challenge for managing MDD is that at least a third of patients experienced inadequate response from antidepressant treatment (17). Pharmacogenetics could help identify the most suitable antidepressant based on an individual's genetic profile on pharmacokinetics and pharmacodynamics (18), though individual genotyping remains expensive. Commercial iTRAQ for discovery of target proteins is also expensive, but if the discovered peripheral target proteins are validated, the cost of measuring only identified peripheral markers becomes more affordable.

In this study, iTRAQ was used to analyze PBMC in patients with MDD, before and after 4 weeks of antidepressant paroxetine, a selective serotonin reuptake inhibitor (SSRI), treatment, in order to identify proteins relevant to paroxetine treatment response.

## Materials and Methods

### Study Samples

The patients were recruited from a tertiary medical center over an 1-year period. Their age was between 20 and 65 years old, and they had no systemic diseases such as diabetes, hypertension, and hyperthyroidism. They also had no smoking or alcohol addiction. All participants had the ability to sign the informed consent, after the investigators thoroughly explained the details of the study. The diagnosis of MDD was established by the same senior psychiatrist, according to the Diagnostic and Statistical Manual of Mental Disorders (DSM)-5 criteria. Patients were free of antidepressants or other medications for at least 1 month prior to entering the study. The severity of MDD was assessed with 17-item Hamilton Depression Rating Scale (HAM-D) (19). All patients had scores greater or equal to 18 prior to treatment. All patients received 4 weeks of paroxetine treatment. Treatment response was defined by HAM-D score reduction of >50%. PBMC of patients were collected in the morning, after fasting for 6 h, before and after 4 weeks of antidepressant treatment. Only those showing treatment response were analyzed (20–22). Written informed consent was provided by all participants after the content and context of the study was fully explained. The institutional review board (IRB) of Chang Gung Memorial Hospital approved the study design (IRB number 201601596B0).

### Isobaric Tags for Relative and Absolute Quantification Labeling

Equal amounts of samples at baseline were pooled, and equal amounts of the samples after treatment were pooled, to minimize the effect of individual variation. One hundred micrograms of protein was reduced, blocked on cysteine, alkylated, and subsequently digested with trypsin overnight at 37°C. The resulting products were than labeled with isobaric tags using a 4-plex iTRAQ kit. Isobaric tags 114 and 115 were used to label lithium responsive groups before and after treatment, respectively, and isobaric tags 116 and 117 were used to label paroxetine responsive groups before and after treatment, respectively. A similar design decision in which only reagents 114 and 116 were used had been seen before to cut down experimental cost (23).

### Peptide Fractionation

The peptides labeled with different channels of isobaric tags were pooled and desalted by using Sep-Pak C18 cartridges (Waters). The desalted peptides were dried under vacuum and then re-suspended in 0.5% trifluoroacetic acid for further fractionized by reversed phase C18 under high pH condition (Pierce High pH Reversed-Phase Peptide Fractionation Kit, Thermo Fisher Scientific, USA) as manufacturer's suggested protocol. The resulting bound peptides were eluted in 1% ammonia solution with various amounts of acetonitrile (5–50 %) resulting in about 10 fractions. Each eluted fraction was then dried under vacuum and re-suspended in 0.1% formic acid solution for the liquid chromatography coupled with tandem mass spectrometry (LC-MS/MS) analysis.

### Tandem Mass Spectrometry-Based Protein Identification

Tandem mass spectrometry analysis was performed on Q Exactive^TM^ HF mass spectrometer (Thermo Fisher, San Jose, California, USA) in combination with a Thermo Scientific^TM^ UltiMate^TM^ 3000 RSLCnano HPLC system. The peptide mixtures were directly loaded onto a 50-cm analytic column (EASY-Spray™ C18 Column), and separated by a gradient with gradually increased of buffer B (80% acetonitrile in 0.1% formic acid) at a flow rate of 250 nl/min over about 165 min. The peptide spectra were acquired in the positive ion mode with a data-dependent acquisition. The top abundant 15 precursor ions within 375-1,400 m/z scan range were then dynamically selected for further fragmented in high collision dissociation (HCD) mode with normalized collision energy set to 33 ± 1. In Full MS scan, the resolution was set to 60,000 at m/z 200, AGC target to 3e6, maximum inject time to 50 ms. In MS/MS scan, the resolution was set to 15,000, with AGC target to 5e4, and maximum inject time set to 100 ms. The release of dynamic exclusion of the selected precursor ions was set to 20 s.

### Mass Spectrometry-Based Proteomics Data Analysis by Proteome Discoverer Software and Mascot Search Engine

The MS files were uploaded into Proteome Discoverer (version 2.1, Thermo Scientific) to generate peak list for protein identification using Mascot search algorithm (version 2.5, Matrix Science) against Swiss-Prot human protein database. For protein identification, the following parameteres were used for database search: carbamidomethylation at Cys as the fixed modification, oxidation at Met, acetylation at protein N-terminus, iTRAQ-labeled at peptide N-terminus and K residue as dynamic modifications, maximum missing cleavage sites with 2, 10 ppm for MS tolerance, and 0.02 Da for MS/MS tolerance. The acceptable false discovery rate of peptide and protein identifications was set to <1%. A minimum of two unique peptides and quantification with two spectrum ratio counts from the unique peptides were required to improve the confidence of the protein identification. The median value of the spectrum ratios was calculated as protein abundance. The global median normalization was applied to recalculate the protein abundance to generate the normalized protein ratios to reduce the system error from sample preparation in each experiment. The mass spectrometry proteomics data have been deposited to the ProteomeXchange Consortium via the PRIDE (24) partner repository with the dataset identifier PXD028947 and 10.6019/PXD028947.

### Western Blotting Validation

The volume of the loading buffer was one-fourth of the total volume. The proteins were denatured at 95°C for 10 min. Following SDS-PAGE, the proteins were transferred to PVDF membranes. After blocking in 2% BSA in PBST for 1 h at room temperature, the PVDF membrane was incubated for 10 h at 4°C with the following primary antibodies: anti-hydroxypyruvate isomerase (HYI) antibody (Abcam, Cambridge, UK), anti-alpha cardiac muscle 1 actin (ACTC1) antibody (R&D Systems, Minneapolis, MN, USA), anti-eukaryotic translation initiation factor 4H (eIF4H) antibody (Thermo Fisher Scientific, Waltham, MA, USA), anti-adenylate kinase isoenzyme 1 (AK1) antibody (Thermo Fisher Scientific, Waltham, MA, USA), anti-mitochondrial dynamin-like 120 kDa protein (OPA1) antibody (R&D Systems, Minneapolis, MN, USA), and anti-RNA binding motif 8A (RBM8A) antibody (Thermo Fisher Scientific, Waltham, MA, USA). Anti-cytochrome c oxidase (COX IV) antibody was used as controls (25). After three washes with PBST, the membrane was incubated with anti-goat or anti-rabbit secondary antibody (1:10,000) at room temperature for 1 h, and then the membrane was washed another five times with PBST (5 min each). The Western blot bands were photographed with UVP BioSpectrum 810 Imaging System (Thermo Fisher Scientific, Waltham, MA, USA). The relative intensities of the protein bands were calculated with ImageJ software (National Institutes of Health), using background subtraction method (26).

### Protein-Protein Interactions

Protein-protein interactions were analyzed using BioGRID database (27). The interested protein names were inputed in the main page (https://thebiogrid.org/), searching with “By Protein/Gene” and “Homo sapiens.”

### Statistical Analysis of iTRAQ Data

The statistical method utilizing peptide-level intensities of iTRAQ reporter ions was used to determine which proteins are significantly changed (28).

## Results

### Study Participants

We recruited six patients with MDD showing response after 4 weeks of paroxetine treatment. Their demographic data were summarized in Table 1. Samples from six patients were analyzed using iTRAQ-based quantitative proteomics.

**Table 1:** Demographic data of paroxetine responsive patients with MDD.

**Pt. no**.	**Age**	**Sex**	**BMI (kg/m^**2**^)**	**Onset age**	**Duration (years)**	**Paroxetine dose (mg/d)**	**HAM-D before**	**HAM-D after**
1	59	F	22.0	59	0.2	20	28	0
2	46	M	25.3	46	2	20	29	0
3	51	M	20.7	51	0.25	40	30	0
4	21	F	17.0	20	0.1	20	29	10
5	63	F	23.2	63	0.5	40	29	0
6	39	M	15.8	38	1	40	26	10

*BMI, body mass index; HAM-D, 17-item Hamilton Depression Rating Scale score*.

### iTRAQ-Based Quantitative Proteomics

Protein and peptide identifications with false determinate rate (FDR) < 0.01 and with at least one unique peptide screened 2,153 proteins. Seven proteins showed more than two-fold difference before and after treatment, and 62 proteins showed differences less than two-fold (Table 2). Six proteins with significant fold changes and commercially available antibodies were identified for further Western blot validation, and they are: putative hydroxypyruvate isomerase (HYI, fold change −311.80), alpha cardiac muscle 1 actin (ACTC1, fold change −14.27), eukaryotic translation initiation factor 4H (eIF4H, fold change −6.42), adenylate kinase isoenzyme 1 (AK1, fold change −3.64), mitochondrial dynamin-like 120 kDa protein (OPA1, fold change −3.55), and RNA binding motif 8A (RBM8A, fold change 2.000). Their representative MS/MS spectra annotated with *b* and *y* ions identified are shown in Supplementary Figures 1–6.

**Table 2:** Western blot validations of potential biomarkers for paroxetine treatment response in MDD patients.

	**Before paroxetine**	**After paroxetine**	***P***
HYI	0.98 ± 0.51	1.11 ± 0.52	0.013*
ACTC1	1.81 ± 0.39	1.73 ± 0.52	0.333
eIF4H	1.53 ± 0.38	1.68 ± 0.36	0.022*
AK1	0.96 ± 0.24	0.97 ± 0.20	0.721
OPA1	0.84 ± 0.38	0.93 ± 0.45	0.646
RBM8A	1.48 ± 0.35	1.69 ± 0.45	0.022*

*ACTC1, alpha cardiac muscle 1 actin; AK1, adenylate kinase isoenzyme 1; EIF4H, eukaryotic translation initiation factor 4H; HYI, putative hydroxypyruvate isomerase; MDD, major depressive disorder; OPA1, mitochondrial dynamin-like 120 kDa protein; RBM8A, RNA binding motif 8A*.

* *p < 0.05*.

### Western Blot Validation

**Figure 1:**
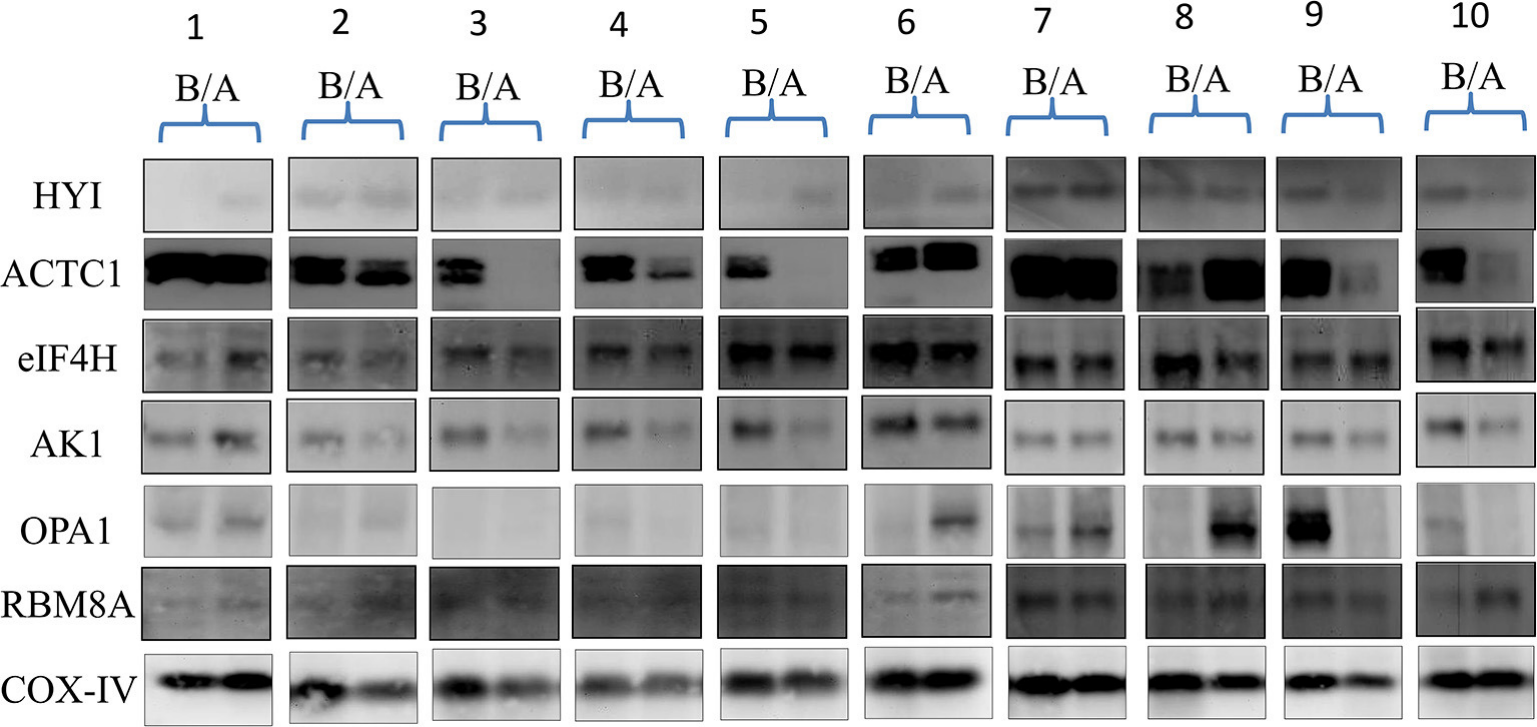
Western blots of 10 MDD patients with paroxetine reponse, before and after antidepressant treatment. A, after treatment; ACTC1, alpha cardiac muscle 1 actin; AK1, adenylate kinase isoenzyme 1; B, before treatment; COX-IV, cytochrome c oxidase subunit IV, as control; EIF4H, eukaryotic translation initiation factor 4H; HYI, putative hydroxypyruvate isomerase; MDD, major depressive disorder; OPA1, mitochondrial dynamin-like 120 kDa protein; RBM8A, RNA binding motif 8A.

Ten pairs of PBMC samples from 10 MDD patients with paroxetine response were analyzed with Western blot analysis (Figure 1). The blot intensities were quantified using ImageJ (Supplementary Table 2). The relative intensities were then calculated with background subtraction method (26) by dividing the intensity of target band by the control (COX-IV) intensity. The relative intensities of HYI, ACTC1, eIF4H, AK1, OPA1, and RBM8A before and after 4 weeks of paroxetine treatment are summarized in Supplementary Table 3. Using paired *t*-test, HYI, eIF4H, and RBM8A showed statistically significant difference before and after paroxetine treatment (*p* = 0.005, 0.019, and 0.013, respectively). Using Wilcoxon signed-rank test, HYI, eIF4H, and RBM8A showed statistically significant difference before and after paroxetine treatment (*p* = 0.013, 0.022, and 0.022, respectively) (Table 2).

### Protein-Protein Interactions

eIF4H (https://thebiogrid.org/113297/summary/homo-sapiens/eif4h.html) showed 88 unique interactors, among them eIF4A1. eIF4A1 (https://thebiogrid.org/108289/summary/homo-sapiens/eif4a1.html) had 241 unique interactors, eIF4A3 included. RBM8a (https://thebiogrid.org/115265/summary/homo-sapiens/rbm8a.html) interacted with eIF4A3, among 171 unique interactors, showing that eIF4H and RBM8a can be linked *via* eukaryotic translation initiation factor family. Those html pages were converted to PDF using a free online converter (https://www.sejda.com/html-to-pdf), and the interested interactors were marked using PDF-XChange Viewer (Version 2.5: Build 322.10). Those html and PDF files were uploaded to a public data depository.

### Peptide-Level Intensities of iTRAQ Reporters

A two-fold cut off was initially used, but after the western blots data we were suggested to evaluate the candidates using a statistical method utilizing peptide-level intensities of iTRAQ reporters to determine which proteins are significantly changed (28). This method could not be applied to HYI, eIF4H, and RBM8A due to their low numbers of peptide spectrum matches (and they all have only one unique peptide), but ACTC1, AK1, and OPA1 all had *p* values lower than 0.05 (*p* = 1.33 x 10^−55^, 0.00162, and 0.0125, respectively). *p*-values for proteins with two-fold increases or decreases were also added to Supplementary Table 1.

## Discussion

The major finding of this study was that by using iTRAQ, we were able to identify three proteins: putative hydroxypyruvate isomerase (HYI), eukaryotic translation initiation factor 4H (eIF4H), and RNA binding motif 8A (RBM8A), which were validated by Western blot, as the potential biomarkers for paroxetine treatment response in MDD patients.

It is the first time HYI and eIF4H were found to associate with psychiatric disorders. HYI catalyzes the reversible isomerization between hydroxypyruvate and 2-hydroxy-3-oxopropanoate (tartronate semialdehyde). HYI had not previously been associated with human diseases. eIF4H, in contrast, had been associated with various diseases, most prominently cancer. In a proteomic investigation of attached (surviving) and detached (dying) cancer cells, eIF4H was identified among six consistently differentially expressed proteins, and silencing it had an anti-proliferative effect (29). In lung adenocarcinoma, eIF4H was the target of miRNA519d to inhibit cell proliferation and invasion (30). eIF4H is overexpressed in lung cancer cells and can be used to predict the response of chemotherapy (31). eIF4h has been found to be associated with neurodegenerative diseases as well. In the post-mortem tissues of patients with amyotrophic lateral sclerosis and frontotemporal degeneration, eIF4H was downregulated in patients with G4C2 mutation compared to patients without the particular mutation and healthy controls, suggesting eIF4H as a disease modifier (32). In a study investigating spatial memory performance and associated transcriptomic alternations in hippocampus of mice underwent chronically unpredictable stress, eIF4H was among identified genes (33). On one hand, while there had been no direct association of HYI and eIF4H with MDD, our findings suggest further investigations would be warranted.

On the other hand, RBM8a had been associated with brain development and animal model of depression. RBM8a is one of the four core proteins of exon junction complex (EJC), along with Magoh, eIF4A3 (DDX48), and CASC3 (MLN51) (34). Microdeletions of 1q21.1 chromosome, which include *Rbm8a* gene, were associated with abnormal brain developments such as microcephaly or macrocephaly (35) as well as moderate mental retardation (36). In an animal study, over-expression of RBM8a in the dentate gyrus of the hippocampus of mice induced abnormal behaviors, in anxiety and depression models such as open field test, social interaction test, and forced swimming test (37). In terms of protein-protein interaction analysis using BioGRID database (27), RBM8a interacts with eIF4A3, which is linked with eIF4A1, which is further connected with eIF4H, suggesting the involvement of eukaryotic translation initiation factor family. Our data suggest RBM8a and eIF4H could be involved in the paroxetine treatment response in MDD patients.

Three other proteins identified through iTRAQ [alpha cardiac muscle 1 actin (ACTC1), adenylate kinase isoenzyme 1 (AK1), and mitochondrial dynamin-like 120 kDa protein (OPA1)] were not verified by the Western blot validations, but they could be linked to MDD in interesting ways.

Mutations of alpha cardiac muscle 1 actin (ACTC1) had been associated with hypertrophic cardiomyopathy (HCM) (38). Studies had shown that patients with HCM were more depressed (39, 40). Furthermore, ACTC1 upregulation could be induced by over-expression of dysbindin (41), a protein found to be reduced in hippocampus in patients with schizophrenia (42, 43). Those data suggested ACTC1 could be affected in psychiatric disorders.

Adenylate kinase (AK) plays a crucial role in energetic metabolism (44). Its isoenzyme AK1 had been found to associate with neurodegenerative diseases. Upregulation of AK1 in the frontal cortex was associated with stages 5-6 of Parkinson's disease (45). Immunization of recombinant AK1 in mice could induce Th1-response as an immune protection (46). MDD had been associated with an activation of the immune/inflammatory system, including changes in serum acute phase protein (47–50) and cytokine levels (51–55). Our previous data also showed that cytokine levels differed between antidepressant responders and non-responders (56). The role of AK1 in MDD treatment response should be followed up in the future.

Mitochondrial dynamin-like 120 kDa protein (OPA1) is a GTPase that regulates mitochondrial fusion and cristae structure in the inner mitochondrial membrane (57). Mutation of OPA1 gene has long been associated with dominant optic atrophy (58), but neurodegenerative symptoms had also been reported, as such parkinsonism and dementia (59), multiple sclerosis-like disorder (60), and fatal infantile mitochondrial encephalomyopathy (61). Disturbances in mitochondrial structure and function could impair neuroplasticity (62), while we did not find any report directly linking OPA1 to MDD at the moment, but a follow-up with more clinical samples could be interesting.

Proteomics had been used in identifying novel biomarkers for MDD, mostly comparing the proteomic profiles of patients with MDD with healthy controls (63). Proteomic studies focusing on antidepressant response were far more scarce, let alone ones focusing on one particular drug (64). Utilization of iTRAQ was also relatively novel. In animal model of depression, iTRAQ analysis identified 241 differentially expressed proteins in hippocampi after Chinese herbal medicine xiaochaihutang (XCHT) treatment, and protein-protein interaction network analysis predicted cell division cycle and apoptosis regulator protein 1 (Ccar1) and Calretinin (Calb2) might play the central roles in XCHT's antidepressant network (65). Our investigation could be the first to use iTRAQ to identify differentially expressed proteins in paroxetine response in patients with MDD.

There were several limitations in our study. The study sample size was small. Samples from six paroxetine responsive patients with MDD were analyzed in the iTRAQ analysis, and only 10 were analyzed in the Western blot validation. Ideally, an independent large cohort should be used to validate the results obtained from iTRAQ analysis, and further analyses such as receiver operating characteristic (ROC) analysis and principal component analysis (PCA) should be performed. Secondly, while significant changes were shown in both iTRAQ and Western blot validation, the directions of changes were not always consistent, which may indicate the possibility of false positive results. Thirdly, only one iTRAQ experiment was performed to compare data before and after paroxetine treatment without experimental, biological, or technical replicate. At least one biological replcate was highly recommended in iTRAQ experiments to minimize variations (66), though by combining our samples we hoped to reduce random biological variations. Fourthly, *p*-values calculation should be performed prior to candidate selection, and proteins identified and quantified by a single peptide are often discarded from further analysis. The readers are warned against over-interpret our study results because of those limitations, and a larger sample size with more controlled variables will be needed before a firm conclusion could be made.

iTRAQ could rapidly identify and quantify differentially expressed proteins, despite being costly. In patients with MDD showing response to paroxetine treatment, putative hydroxypyruvate isomerase (HYI), eukaryotic translation initiation factor 4H (eIF4H), and RNA binding motif 8A (RBM8A) were differentially expressed before and after treatment, as identified by iTRAQ and validated by Western blot. Those identified proteins could be potential predictors of paroxetine treatment response in patients with MDD. This finding could help establish future individualized medicine.

## Data Availability Statement

The mass spectrometry proteomics data have been deposited to the ProteomeXchange Consortium *via* the PRIDE (24) partner repository with the dataset identifier PXD028947 and 10.6019/PXD028947.

## Ethics Statement

The studies involving human participants were reviewed and approved by the Institutional Review Board (IRB) of Chang Gung Memorial Hospital. The patients/participants provided their written informed consent to participate in this study.

## Author Contributions

T-LH and JS contributed substantially to conception and design. T-LH and HS contributed to acqusition of data. HS, C-CL, and T-LH helped with analysis and interpretation of data. C-CL drafted the article and revised it critically for important intellectual content. T-LH approved the final draft. All authors contributed to the article and approved the submitted version.

## Funding

This work was supported by a clinical research grants CMRPG8G1161 and CMRPG8J1511 from Kaohsiung Chang Gung Memorial Hospital, Taiwan and Ministry of Science and Technology, Taiwan (NMRPG8H0101).

## Conflict of Interest

The authors declare that the research was conducted in the absence of any commercial or financial relationships that could be construed as a potential conflict of interest.

## Publisher's Note

All claims expressed in this article are solely those of the authors and do not necessarily represent those of their affiliated organizations, or those of the publisher, the editors and the reviewers. Any product that may be evaluated in this article, or claim that may be made by its manufacturer, is not guaranteed or endorsed by the publisher.
