# Corrigendum: Isobaric Tags for Relative and Absolute Quantitation Identification of Blood Proteins Relevant to Paroxetine Response in Patients With Major Depressive Disorder

**DOI:** 10.3389/fpsyt.2022.937238

**Published:** 2022-07-06

**Authors:** Chin-Chuen Lin, Hung Su, Jentaie Shiea, Tiao-Lai Huang

**Affiliations:** ^1^Department of Psychiatry, Kaohsiung Chang Gung Memorial Hospital, Chang Gung University College of Medicine, Kaohsiung, Taiwan; ^2^Department of Chemistry, National Sun Yat-Sen University, Kaohsiung, Taiwan; ^3^Genomic and Proteomic Core Laboratory, Department of Medical Research, Kaohsiung Chang Gung Memorial Hospital, Kaohsiung, Taiwan

**Keywords:** eukaryotic translation initiation factor 4H (eIF4H), iTRAQ, major depressive disorder, putative hydroxypyruvate isomerase (HYI), RNA binding motif 8A (RBM8A)

In the original article, there was an error. A typo was present in the IRB number. A correction has been made to **Materials and Methods**, “**Study Samples**,” paragraph one.

“The institutional review board (IRB) of Chang Gung Memorial Hospital approved the study design (IRB number 201501596B0)” has been corrected to “The institutional review board (IRB) of Chang Gung Memorial Hospital approved the study design (IRB number 201601596B0).”

The authors apologize for this error and state that this does not change the scientific conclusions of the article in any way. The original article has been updated.

## Publisher's Note

All claims expressed in this article are solely those of the authors and do not necessarily represent those of their affiliated organizations, or those of the publisher, the editors and the reviewers. Any product that may be evaluated in this article, or claim that may be made by its manufacturer, is not guaranteed or endorsed by the publisher.

