# Supplementary material for: Isobaric Tags for Relative and Absolute Quantitation Identification of Blood Proteins Relevant to Paroxetine Response in Patients With Major Depressive Disorder
Source: Front Psychiatry. 2022 Apr 18;13:577857. doi: 10.3389/fpsyt.2022.577857 (PMC9058070; doi:10.3389/fpsyt.2022.577857)
Supplement: Supplementary file 1 [file Data_Sheet_1.pdf]

Supplementary Table 1. Differentially expressed proteins before and after paroxetine response in patients with MDD

Accession	Description	gene name	MW [kDa]	Fold change T/N	*p*
Q5T013	**Putative hydroxypyruvate isomerase**	**HYI**	30.4	**-311.80**	N/A ↓
P63261	Actin, cytoplasmic 2	ACTG1	41.8	-19.41	$1.06x10^{-71}$ ↓
P68032	**Actin, alpha cardiac muscle 1**	**ACTC1**	42	**-14.27**	$1.33x10^{-55}$ ↓
Q15056	**Eukaryotic translation initiation factor 4H**	**EIF4H**	27.4	**-6.42**	N/A ↓
O14950	Myosin regulatory light chain 12B	MYL12B	19.8	-6.10	$2.5x10^{-20}$ ↓
Q9UHA4	Ragulator complex protein LAMTOR3	LAMTOR3	13.6	-5.51	N/A ↓
P0C0S5	Histone H2A.Z	H2AFZ	13.5	-4.46	$2.59x10^{-9}$ ↓
Q6ZUX7	Lipoma HMGIC fusion partner-like 2 protein	LHFPL2	24.5	-4.15	N/A ↓
P30044	Peroxiredoxin-5, mitochondrial	PRDX5	22.1	-4.07	$4.21x10^{-16}$ ↓
P16104	Histone H2AX	H2AFX	15.1	-4.06	$2.91x10^{-8}$ ↓
P61960	Ubiquitin-fold modifier 1	UFM1	9.1	-4.02	0.036453 ↓
Q92747	Actin-related protein 2/3 complex subunit 1A	ARPC1A	41.5	-3.71	0.031932 ↓
P63313	Thymosin beta-10	TMSB10	5	-3.70	$6.69x10^{-6}$ ↓
P00568	**Adenylate kinase isoenzyme 1**	**AK1**	21.6	**-3.64**	0.001616 ↓
O60313	**Dynamin-like 120 kDa protein, mitochondrial**	**OPA1**	111.6	**-3.55**	0.012513 ↓
P21291	Cysteine and glycine-rich protein 1	CSRP1	20.6	-3.50	0.000724 ↓
Q9H6Q3	Src-like-adapter 2	SLA2	28.6	-3.48	N/A ↓
Q86YW5	Trem-like transcript 1 protein	TREML1	32.7	-3.48	0.011018 ↓

P01111	GTPase NRas	NRAS	21.2	-3.42	0.004407 ↓
P32189	Glycerol kinase	GK	61.2	-3.37	0.050401 ↓
O14776	Transcription elongation regulator 1	TCERG1	123.8	-3.00	N/A ↓
P31153	S-adenosylmethionine synthase isoform type-2	MAT2A	43.6	-2.93	0.085565 ↓
P30041	Peroxiredoxin-6	PRDX6	25	-2.90	$5.11x10^{-14}$ ↓
P10809	60 kDa heat shock protein, mitochondrial	HSPD1	61	-2.85	$1.2\ x10^{-7}$ ↓
P68366	Tubulin alpha-4A chain	TUBA4A	49.9	-2.84	$6.11x10^{-21}$ ↓
P11177	Pyruvate dehydrogenase E1 component subunit beta, mitochondrial	PDHB	39.2	-2.79	0.000213 ↓
O14979	Heterogeneous nuclear ribonucleoprotein D-like	HNRNPDL	46.4	-2.79	0.355557 ↓
P60660	Myosin light polypeptide 6	MYL6	16.9	-2.75	$4.79x10^{-15}$ ↓
P22102	Trifunctional purine biosynthetic protein adenosine-3	GART	107.7	-2.73	0.001732 ↓
P14618	Pyruvate kinase PKM	PKM	57.9	-2.72	$1.5\ x10^{-26}$ ↓
P09382	Galectin-1	LGALS1	14.7	-2.71	$1.47\ x10^{-7}$ ↓
P43686	26S protease regulatory subunit 6B	PSMC4	47.3	-2.68	0.030568 ↓
P04406	Glyceraldehyde-3-phosphate dehydrogenase	GAPDH	36	-2.66	$4.03x10^{-30}$ ↓
Q9ULC5	Long-chain-fatty-acid--CoA ligase 5	ACSL5	75.9	-2.66	N/A ↓
P15170	Eukaryotic peptide chain release factor GTP-binding subunit ERF3A	GSPT1	55.7	-2.59	0.073919 ↓
P63241	Eukaryotic translation initiation factor 5A-1	EIF5A	16.8	-2.58	$3.68x10^{-5}$ ↓
O14579	Coatomer subunit epsilon	CO	34.5	-2.58	0.000644 ↓
P24666	Low molecular weight phosphotyrosine protein phosphatase	ACP1	18	-2.58	0.027367 ↓

P57764	Gasdermin-D	GSDMD	52.8	-2.51	0.000325 ↓
P06576	ATP synthase subunit beta, mitochondrial	ATP5B	56.5	-2.50	$7.18x10^{-14}$ ↓
Q9Y6E0	Serine/threonine-protein kinase 24	STK24	49.3	-2.50	0.127583 ↓
O43852	Calumenin	CALU	37.1	-2.50	N/A ↓
P31146	Coronin-1A	CORO1A	51	-2.49	0.000301 ↓
P60709	Actin, cytoplasmic 1	ACTB	41.7	-2.47	$1.41x10^{-72}$ ↓
P49591	Serine--tRNA ligase, cytoplasmic	SARS	58.7	-2.47	0.03702 ↓
Q12965	Unconventional myosin-Ie	MYO1E	127	-2.43	N/A ↓
Q9H3S7	Tyrosine-protein phosphatase non-receptor type 23	PTPN23	178.9	-2.43	0.008297 ↓
Q13884	Beta-1-syntrophin	SNTB1	58	-2.42	0.206161 ↓
P35232	Prohibitin	PHB	29.8	-2.42	0.000441 ↓
Q86TP1	Protein prune homolog	PRUNE	50.2	-2.41	0.030335 ↓
P40939	Trifunctional enzyme subunit alpha, mitochondrial	HADHA	82.9	-2.40	0.001922 ↓
Q9Y3E5	Peptidyl-tRNA hydrolase 2, mitochondrial	PTRH2	19.2	-2.36	N/A ↓
P00403	Cytochrome c oxidase subunit 2	MT-CO2	25.5	-2.35	0.058764 ↓
Q96HS1	Serine/threonine-protein phosphatase PGAM5, mitochondrial	PGAM5	32	-2.35	0.0218 ↓
Q9NP79	Vacuolar protein sorting-associated protein VTA1 homolog	VTA1	33.9	-2.33	0.142308 ↓
P62857	40S ribosomal protein S28	RPS28	7.8	-2.33	N/A ↓
Q8IWA4	Mitofusin-1	MFN1	84	-2.33	N/A ↓
P22061	Protein-L-isoaspartate(D-aspartate) O-methyltransferase	PCMT1	24.6	-2.30	0.057328 ↓
O00186	Syntaxin-binding protein 3	STXBP3	67.7	-2.29	0.108913 ↓

P55735	Protein SEC13 homolog	SEC13	35.5	-2.29	0.037425 ↓
P24844	Myosin regulatory light polypeptide 9	MYL9	19.8	-2.28	$1.15x10^{-17}$ ↓
Q8N5K1	CDGSH iron-sulfur domain-containing protein 2	CISD2	15.3	-2.25	0.098942 ↓
Q16799	Reticulon-1	RTN1	83.6	-2.250	0.284809 ↓
P53597	Succinyl-CoA ligase [ADP/GDP-forming] subunit alpha, mitochondrial	SUCLG1	36.2	-2.250	0.003078 ↓
P23528	Cofilin-1	CFL1	18.5	-2.250	$5.6x10^{-22}$ ↓
Q9Y5S9	**RNA-binding motif 8A**	**RBM8A**	19.9	**2.000**	N/A ↑
P24158	Myeloblastin	PRTN3	27.8	2.028	$1.38x10^{-11}$ ↑
P25815	Protein S100-P	S100P	10.4	2.041	0.002981 ↑
P80188	Neutrophil gelatinase-associated lipocalin	LCN2	22.6	2.046	$3.2x10^{-19}$ ↑
Q969E4	Transcription elongation factor A protein-like 3	TCEAL3	22.5	2.115	N/A ↑
P20160	Azurocidin	AZU1	26.9	2.264	$9.37x10^{-7}$ ↑
P59665	Neutrophil defensin 1	DEFA1	10.2	2.420	0.00141 ↑

MW: Molecular weight; PBMC: peripheral blood mononuclear cell

Supplementary Table 2. Western blot intensities quantified using ImageJ

Patient	1		2		3		4		5		6		7		8		9		10	
Status	B	A	B	A	B	A	B	A	B	A	B	A	B	A	B	A	B	A	B	A
HYI	204369	228116	182059	184471	181058	177977	211985	188958	235926	217580	236089	336839	263054	330218	261107	502742	539039	240779	303797	210245
ACTC1	529676	530316	509251	466306	508336	343523	544472	431397	459290	334386	589700	658732	572460	572535	627669	703482	662994	437708	625607	452201
eIF4H	295599	322632	366660	453574	532913	523620	505702	535835	544047	511944	389596	375427	481256	466220	562724	564400	515658	493281	455377	496639
AK1	98826	122329	148309	172952	210807	217662	240277	266875	271545	319062	396007	368483	298594	314193	487832	470727	456430	443228	416879	399381
OPA1	297836	363736	345442	357352	492092	492041	513678	525290	595640	577231	595090	571573	402851	399538	546387	491250	493738	495090	525290	487508
RBM8A	235294	258298	218284	199630	319674	289127	354793	336026	401366	345104	418602	382683	208599	207015	277737	249575	270390	251104	306199	270070
HYI	274983	324716	340768	268131	351493	282490	294675	266197	346029	321168	352563	354239	389462	351011	269161	232800	305245	253035	260607	247498
COX-IV	204369	228116	182059	184471	181058	177977	211985	188958	235926	217580	236089	336839	263054	330218	261107	502742	539039	240779	303797	210245

A: after treatment; ACTC1: alpha cardiac muscle 1 actin; AK1: adenylate kinase isoenzyme 1; B: before treatment; COX-IV: cytochrome c oxidase subunit IV, as control; EIF4H: eukaryotic translation initiation factor 4H; HYI: putative hydroxypyruvate isomerase; MDD: major depressive disorder; OPA1: mitochondrial dynamin-like 120 kDa protein; RBM8A: RNA binding motif 8A

Supplementary Table 3. Relative intensities of western blot bands

Patient	1		2		3		4		5		6		7		8		9		10	
Status	B	A	B	A	B	A	B	A	B	A	B	A	B	A	B	A	B	A	B	A
HYI	0.3594	0.3767	0.4352	0.6450	0.5997	0.7705	0.8154	1.0025	0.7847	0.9934	1.1232	1.0402	0.7667	0.8951	1.8124	2.0220	1.4953	1.7516	1.5996	1.6137
ACTC1	1.9262	1.6332	1.4944	1.7391	1.4462	1.2161	1.8477	1.6206	1.3273	1.0412	1.6726	1.8596	1.4699	1.6311	2.3319	3.0218	2.1720	1.7298	2.4006	1.8271
eIF4H	1.0831	1.1202	1.0137	1.3328	1.4000	1.7418	1.7432	1.9733	1.7214	1.7973	1.6879	1.6135	1.0344	1.1382	2.0300	2.1102	1.6175	1.9566	2.0156	1.9697
AK1	0.8557	0.7955	0.6406	0.7445	0.9095	1.0235	1.2040	1.2623	1.1599	1.0745	1.1873	1.0803	0.5356	0.5898	1.0319	1.0721	0.8858	0.9924	1.1749	1.0912
OPA1	0.7432	0.7025	0.5343	0.6880	0.5151	0.6300	0.7194	0.7098	0.6818	0.6775	0.6696	0.9509	0.6754	0.9408	0.9701	2.1595	1.7659	0.9516	1.1657	0.8495
RBM8A	1.0750	0.9936	1.0760	1.6916	1.5161	1.8536	1.7161	2.0129	1.5723	1.5940	1.1050	1.0598	1.2357	1.3282	2.0907	2.4244	1.6893	1.9495	1.7474	2.0066
HYI	0.3594	0.3767	0.4352	0.6450	0.5997	0.7705	0.8154	1.0025	0.7847	0.9934	1.1232	1.0402	0.7667	0.8951	1.8124	2.0220	1.4953	1.7516	1.5996	1.6137

A: after treatment; ACTC1: alpha cardiac muscle 1 actin; AK1: adenylate kinase isoenzyme 1; B: before treatment; EIF4H: eukaryotic translation initiation factor 4H; HYI: putative hydroxypyruvate isomerase; MDD: major depressive disorder; OPA1: mitochondrial dynamin-like 120 kDa protein; RBM8A: RNA binding motif 8A

Supplementary Figure 1. Representative MS/MS spectrum annotated with *b* and *y* ions identified for HYI

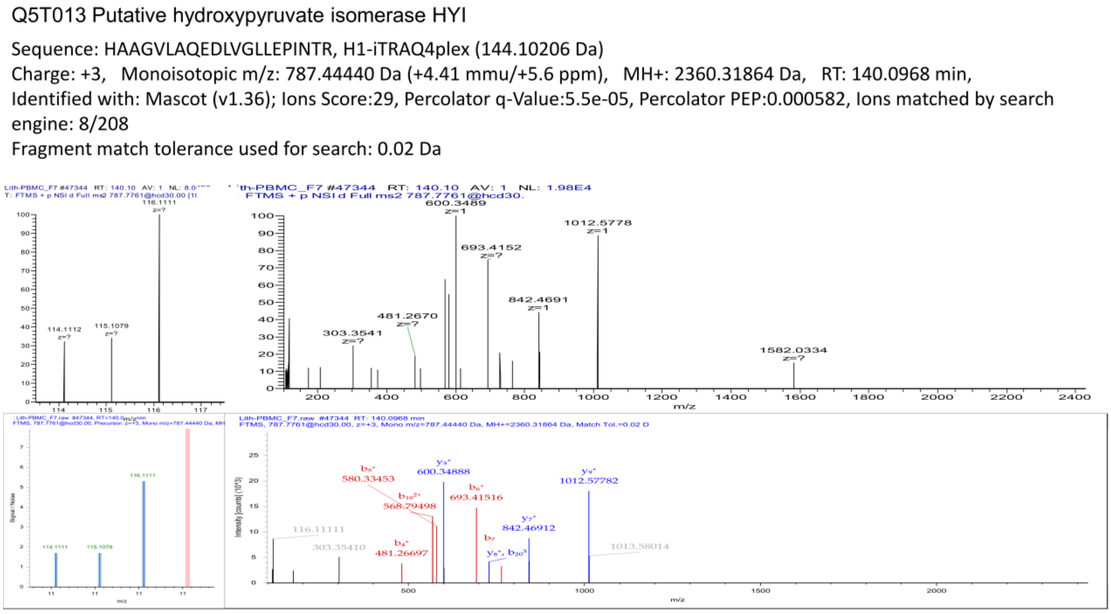

HYI: putative hydroxypyruvate isomerase

Supplementary Figure 2. Representative MS/MS spectrum annotated with *b* and *y* ions identified for ACTC1

P68032 Actin, alpha cardiac muscle 1 ACTC1
Sequence: YPIEHGIITNWDDMEK, K16-iTRAQ4plex (144.10206 Da), Y1-iTRAQ4plex (144.10206 Da), M14-Oxidation (15.99492 Da)
Charge: +3, Monoisotopic m/z: 755.71021 Da (+2.03 mmu/+2.69 ppm), MH+: 2265.11606 Da, RT: 131.2353 min, Identified with: Mascot (v1.36); Ions Score:24, Percolator q-Value:0, Percolator PEP:0.000253, Ions matched by search engine: 6/154
Fragment match tolerance used for search: 0.02 Da

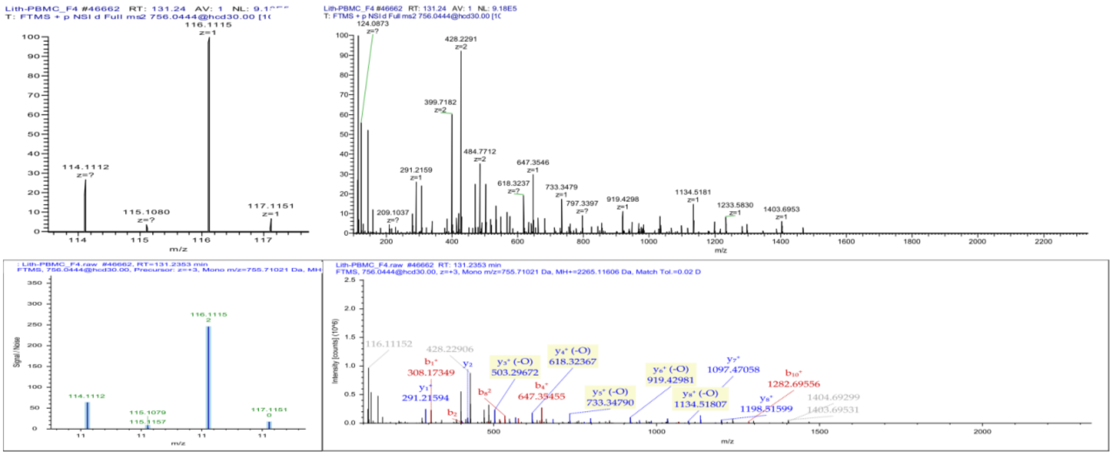

ACTC1: alpha cardiac muscle 1 actin

Supplementary Figure 3. Representative MS/MS spectrum annotated with *b* and *y* ions identified for eIF4H

Q15056 Eukaryotic translation initiation factor 4H EIF4H

Sequence: EALTYDGALLGDR, E1-iTRAQ4plex (144.10206 Da)
Charge: +2, Monoisotopic m/z: 769.40576 Da (+3.2 mmu/+4.16 ppm), MH+: 1537.80425 Da, RT: 119.2028 min,
Identified with: Mascot (v1.36); Ions Score:49, Percolator q-Value:0, Percolator PEP:0.000286, Ions matched by search engine: 6/118
Fragment match tolerance used for search: 0.02 Da

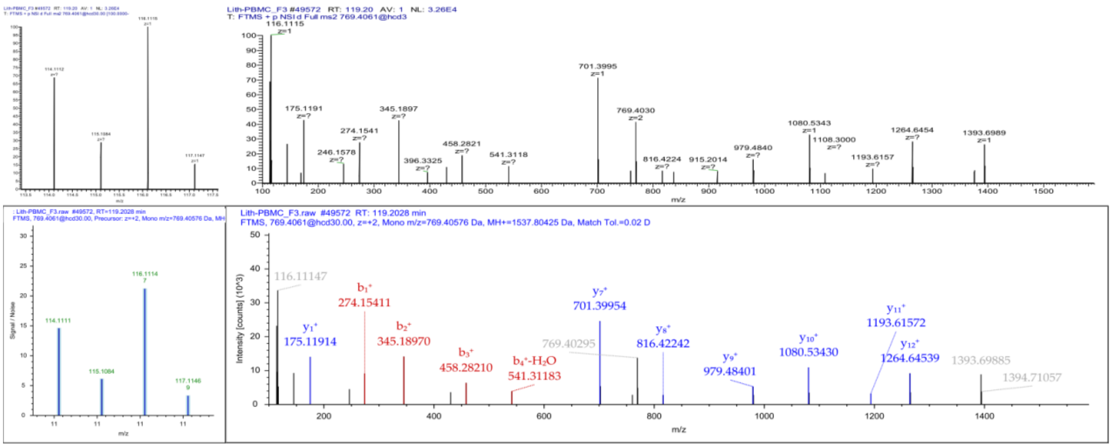

eIF4H: eukaryotic translation initiation factor 4H

Supplementary Figure 4. Representative MS/MS spectrum annotated with *b* and *y* ions identified for AK1

P00568 Adenylate kinase isoenzyme 1 AK1
Sequence: IIFVVGGPGSGK, K12-iTRAQ4plex (144.10206 Da), I1-iTRAQ4plex (144.10206 Da)
Charge: +2, Monoisotopic m/z: 709.93701 Da (+2.9 mmu/+4.09 ppm), MH+: 1418.86675 Da, RT: 92.7591 min,
Identified with: Mascot (v1.36); Ions Score:100, Percolator q-Value:0, Percolator PEP:6.07e-08, Ions matched by search engine: 11/88
Fragment match tolerance used for search: 0.02 Da

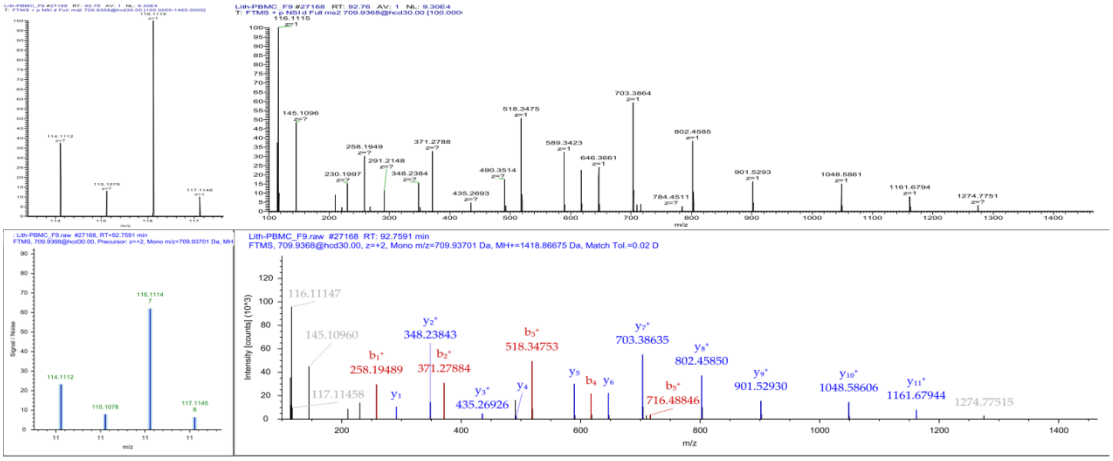

AK1: adenylate kinase isoenzyme 1

Supplementary Figure 5. Representative MS/MS spectrum annotated with *b* and *y* ions identified for OPA1

O60313 Dynamin-like 120 kDa protein, mitochondrial OPA1

Sequence: IQQIIEGK, K8-iTRAQ4plex (144.10206 Da), I1-iTRAQ4plex (144.10206 Da)
Charge: +2, Monoisotopic m/z: 608.88043 Da (+1.63 mmu/+2.67 ppm), MH+: 1216.75359 Da, RT: 57.4136 min,
Identified with: Mascot (v1.36); Ions Score:33, Percolator q-Value:0.00174, Percolator PEP:0.0107, Ions matched by search engine: 5/68
Fragment match tolerance used for search: 0.02 Da

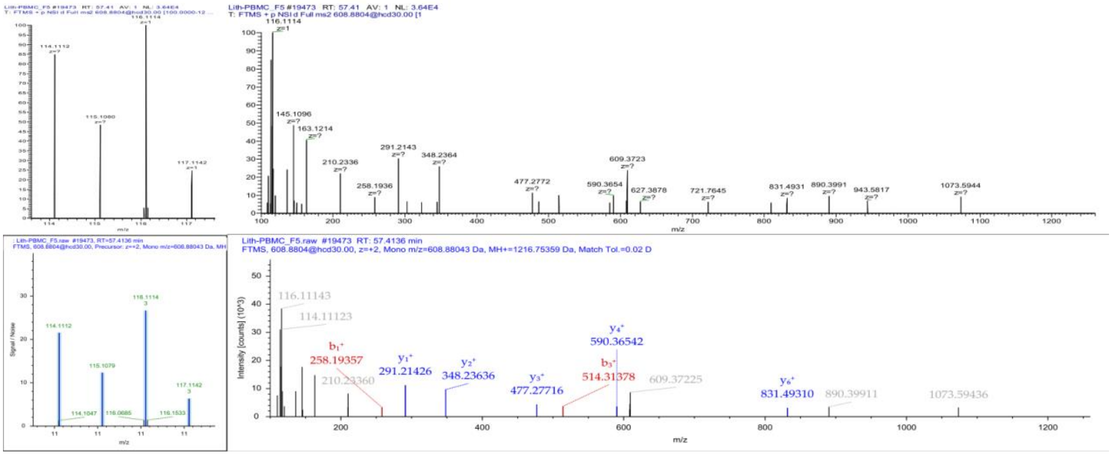

OPA1: mitochondrial dynamin-like 120 kDa protein

Supplementary Figure 6. Representative MS/MS spectrum annotated with *b* and *y* ions identified for RBM8A

Q9Y5S9 RNA-binding motif 8A RBM8A
Sequence: MREDYDSVEQDGDEPGPQR, M1-iTRAQ4plex (144.10206 Da), M1-Oxidation (15.99492 Da)
Charge: +3, Monoisotopic m/z: 795.01416 Da (+1.82 mmu/+2.3 ppm), MH+: 2383.02793 Da, RT: 140.4700 min,
Identified with: Mascot (v1.36); Ions Score:56, Percolator q-Value:0, Percolator PEP:9.84e-07, Ions matched by search engine: 10/200
Fragment match tolerance used for search: 0.02 Da

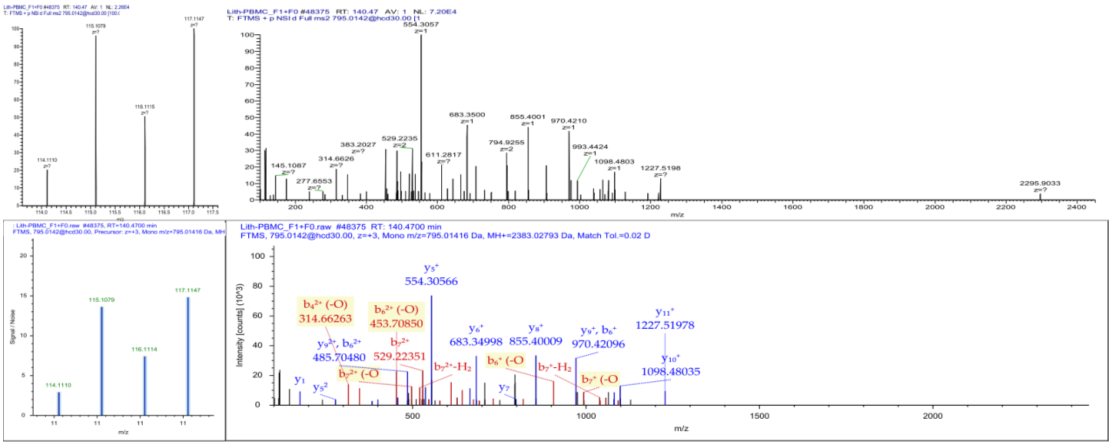

RBM8A: RNA binding motif 8A
